# Browning of white adipose tissue after a burn injury promotes hepatic steatosis and dysfunction

**DOI:** 10.1038/s41419-019-2103-2

**Published:** 2019-11-18

**Authors:** Abdikarim Abdullahi, Osai Samadi, Christopher Auger, Tharsan Kanagalingam, Darren Boehning, Sheng Bi, Marc G. Jeschke

**Affiliations:** 1 Faculty of Medicine, University of Toronto, Toronto, ON Canada; 2 Biological Sciences, Sunnybrook Research Institute, Toronto, ON Canada; 3 Department of Biochemistry and Molecular Biology, University of Texas Health Science Center at Houston, Houston, TX USA; 4 Department of Psychiatry and Behavioral Sciences, Johns Hopkins University School of Medicine, Baltimore, MD USA; 5 Ross Tilley Burn Centre, Sunnybrook Hospital, Toronto, ON Canada; 6 Department of Surgery, Division of Plastic Surgery and Department of Immunology, University of Toronto, Toronto, ON Canada

**Keywords:** Molecular biology, Experimental models of disease

## Abstract

Burn patients experiencing hypermetabolism develop hepatic steatosis, which is associated with liver failure and poor outcomes after the injury. These same patients also undergo white adipose tissue (WAT) browning, which has been implicated in mediating post-burn cachexia and sustained hypermetabolism. Despite the clinical presentation of hepatic steatosis and WAT browning in burns, whether or not these two pathological responses are linked remains poorly understood. Here, we show that the burn-induced WAT browning and its associated increased lipolysis leads to the accelerated development of hepatic steatosis in mice. Deletion of interleukin 6 (IL-6) and the uncoupling protein 1 (UCP1), regulators of burn-induced WAT browning completely protected mice from hepatic steatosis after the injury. Treatment of post-burn mice with propranolol or IL-6 receptor blocker attenuated burn-induced WAT browning and its associated hepatic steatosis pathology. Lipidomic profiling in the plasma of post-burn mice and burn patients revealed elevated levels of damage-inducing lipids (palmitic and stearic acids), which induced hepatic endoplasmic reticulum (ER) stress and compromised hepatic fat oxidation. Mechanistically, we show that hepatic ER stress after a burn injury leads to a greater ER-mitochondria interaction, hepatocyte apoptosis, oxidative stress, and impaired fat oxidation. Collectively, our findings uncover an adverse “cross-talk” between the adipose and liver tissue in the context of burn injury, which is critically mediated by WAT browning.

## Introduction

Hypermetabolic reprogramming is considered a hallmark of burns and has been an area of accelerated research over the last decade. A common theme emerging from this work is that when nutrients are scarce, metabolic signaling pathways direct enhanced nutrient acquisition via the mobilization of lipids and proteins^1,2^. This mobilization may result in cachexia, which is associated with the loss of muscle proteins, and alterations in fat metabolism including a substantial induction of lipolysis. While muscle catabolism and hepatic steatosis are well documented in the pathophysiology of burn injury, the involvement of the white adipose tissue (WAT) in this process is essentially unknown. Recently, however, two independent studies related to the metabolic consequences of cancer and burns described the phenomenon of WAT browning in human and rodent models, in which subcutaneous white adipocytes convert to a phenotypically similar brown-like adipocyte, termed beige/brite adipose tissue^3–5^.

During burn-induced WAT browning, systemic elevations in catecholamines lead to the activation of the β_3_-adrenergic receptor, which stimulates lipolysis and subsequently increases the expression of UCP1 within white adipocytes^4,6,7^. Of great importance is the link between WAT lipolysis and browning. Lipolysis is almost ubiquitously present in burn patients and has been associated with increased insulin resistance, and most importantly, fatty acid infiltration in essential organs, such as the liver and kidney^8–10^. Furthermore, in both cancer patients and patients suffering from cardiovascular diseases, browning of WAT and its associated increased lipolytic state has been implicated in facilitating organ steatosis (muscle and cardiac tissues)^3,5,11^. The possibility of the latter scenario has not been investigated in the context of burn injury, especially given that relative to the skeletal and cardiac muscle, the liver is the major organ mobilized to respond to lipids emanating from the adipose tissue after the injury.

Despite this association between the lipolysis and hepatic steatosis post-burn injury, no studies have evaluated the altered lipid molecules and aberrant lipid metabolites that infiltrate the liver to induced hepatic dysfunction. Indeed, hepatic steatosis-associated dysfunction in the liver remains a leading cause of mortality in burn patients^9^. Significant evidence from epidemiological and observational studies have found that burn patients with pre-existing liver disease not only have increased mortality risk from 6 to 27% of the total population, but post-mortem liver analyses have determined that patients who succumb to the injury demonstrate significant hepatic steatosis and hepatomegaly^12^.

In this study, we investigated the impact of burn-induced WAT browning and its associated lipolysis on hepatic steatosis. Our findings show that burn-induced browning leads to the development of hepatic steatosis post-burn injury. Inhibition of the upstream regulator (IL-6) and the downstream regulator (UCP-1) of burn-induced browning protected mice from the development of hepatic steatosis. Lipidomic analysis in samples taken from both burn patients and post-burn mice revealed the expression of stress-inducing lipids palmitic and stearic acids, which resulted in both hepatic ER stress and impaired fat oxidation in hepatocytes. Thus, our findings establish an important link between WAT browning and hepatic steatosis, which provides a novel target to ameliorate hepatic steatosis and its associated cellular changes after a burn injury.

## Results

### WAT browning is associated with lipid accumulation in the livers of post-burn mice

**Fig. 1:**
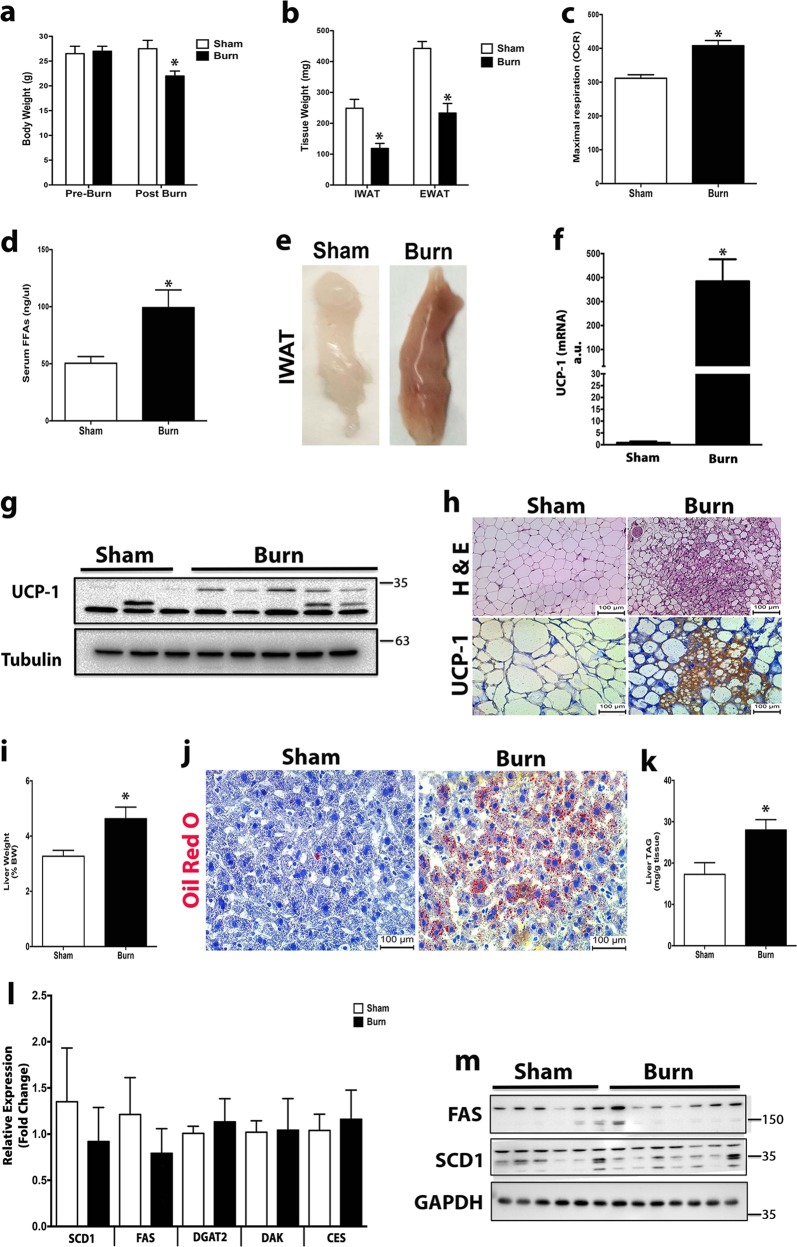
Browning of white adipose tissue leads to the development of hepatic steatosis post-burn injury. **a**, **b** Changes in total body (**a**) and adipose tissue (**b**) weight in post-burn and control mice. **c** Analysis of oxygen consumption rate in isolated inguinal WAT of post burned mice and controls. **d** Plasma concentration of free fatty acids in burned mice and controls. **e** Representative macroscopic pictures of isolated inguinal WAT from burned mice and controls at autopsy. **f** Quantitative RT-PCR analysis of browning gene Uncoupling protein 1 (UCP1) in inguinal WAT of burned mice and controls. **g** Immunoblot analysis of UCP1 in inguinal WAT of burned mice and controls. **h** H&E and UCP1 staining in inguinal WAT of burned mice and controls. **i** Liver weights normalized to body weight of burned mice and controls. **j** Oil Red O staining for fat droplets in liver sections from burned mice and controls. **k** Triglyceride (TG) content of livers from burned mice and controls. **l** Quantitative RT-PCR analysis of lipogenic genes was measured in livers from burned mice and controls. **m** Immunoblot analysis of lipogenic proteins in livers from burned mice and controls. Data represented as mean ± SEM, *p* < 0.05 *significant difference burn vs. controls (*n* = 8, biological replicates, experiments repeated two times).

In order to investigate the relationship between burn-induced WAT browning and hepatic steatosis, we utilized a mouse model of burn injury. Mice were subjected to a 30% total body surface area (TBSA) thermal injury, which successfully induced a hypermetabolic response characterized by body weight loss and accelerated wasting of inguinal WAT (iWAT) and epididymal WAT (eWAT) (Fig. 1a, b). Additionally, the rate of oxygen consumption and lipolysis were significantly elevated in post-burn mice (Fig. 1c, d). This hypermetabolic response to burn injury was accompanied by the stimulation of the browning process, as previously reported in burn patients, namely a phenotypic switch from white to beige fat in subcutaneous iWAT (Fig. 1e), elevated gene and protein expression of the key browning marker UCP1 (Fig. 1f, g) and an increased presence of multilocular cells and UCP1-positive histological staining (Fig. 1h).

Interestingly, the aforementioned changes in the adipose tissue were also associated with alterations in the liver of post-burn mice. In fact, we observed a marked increase in liver weight relative to body weight of burned mice compared to controls (Fig. 1i). Histologically, livers from burned mice exhibited substantially greater lipid accumulation compared to those of control mice (Fig. 1j). Mirroring the histological findings, burned mice exhibited almost a 2-fold increase in hepatic TG content compared to control mice (Fig. 1k). To further link the fatty acid accumulation in the liver of post-burn mice to alterations in the adipose tissue and not hepatic *de novo* lipogenesis (DNL), we measured the expression of key genes associated with DNL. Hepatic expression of key DNL genes (*Scd1, Fas, Dgat2, Dak, Ces*) were not significantly upregulated in response to burn injury compared to control mice (Fig. 1l). Corroborating our gene expression data, we found no significant upregulation of key DNL proteins FAS and SCD1 (Fig. 1m). Together, these findings suggest that the hepatic steatosis observed post-burn injury is a result of the changes in the adipose tissue rather than an increase in hepatic DNL.

### Essential roles of IL-6 and UCP1 in mediating burn-induced browning and hepatic steatosis

**Fig. 2:**
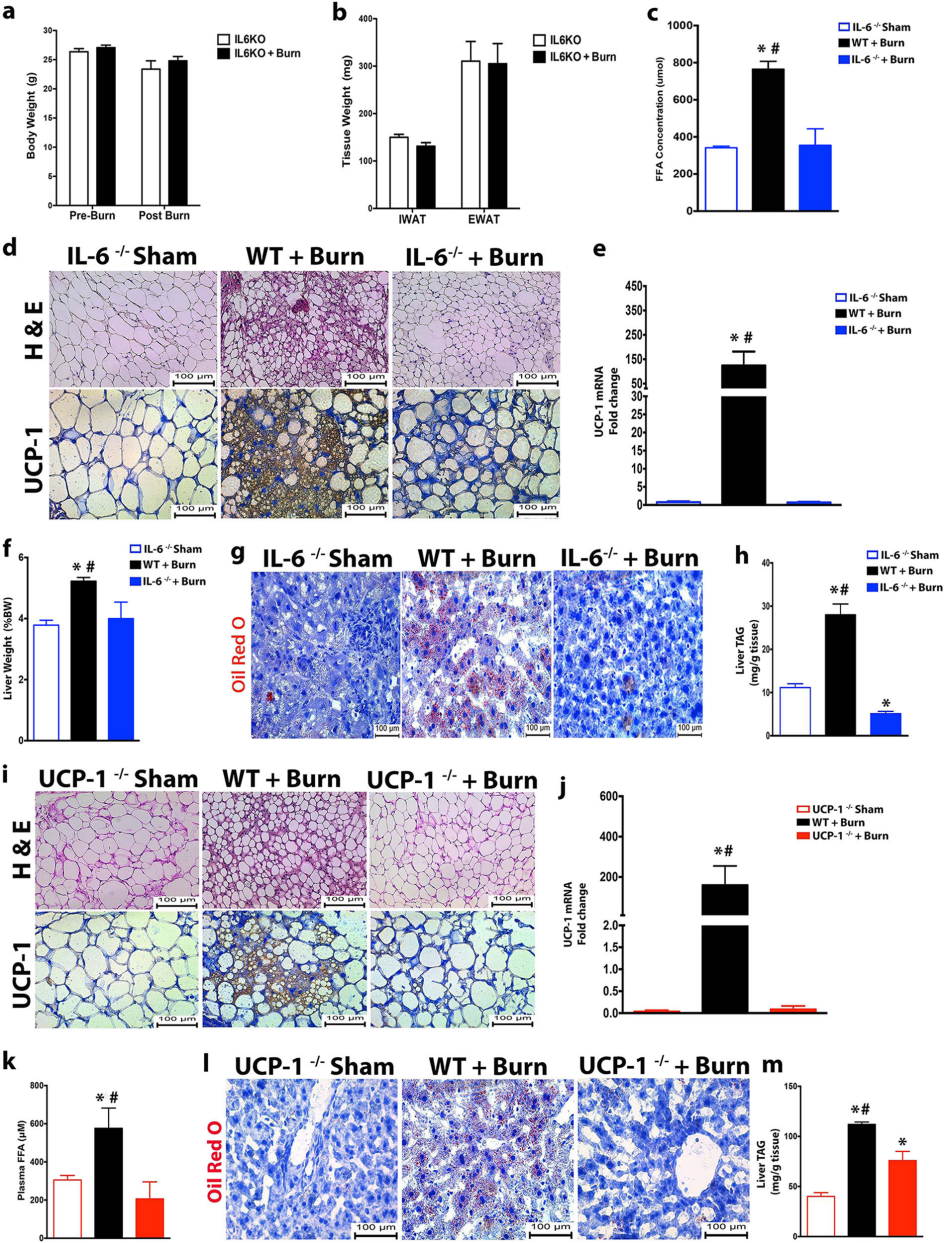
IL-6^−/−^ and UCP-1^−/−^ KO mice are protected from burn-induced browning and hepatic steatosis post-injury. **a**, **b** Changes in total body (**a**) and fat (**b**) weight in IL-6^−/−^ burned mice and IL-6^−/−^ controls. **c** Plasma concentration of free fatty acids in IL-6^−/−^ burned mice and controls. **d** UCP1 staining in inguinal WAT of WT and IL-6^−/−^ burned mice and controls. **e** Quantitative RT-PCR analysis of browning gene UCP1 in inguinal WAT of wild type (WT) and IL-6^−/−^ burned mice and controls. **f** Liver weights normalized to body weight of WT and IL-6^−/−^ burned mice and controls. **g** Oil Red O staining for fat droplets in liver sections from WT and IL-6^−/−^ burned mice and controls. **h** Triglyceride (TG) content of livers from WT and IL-6^−/−^ burned mice and controls. **i** H&E and UCP1 staining in inguinal WAT of WT and UCP-1^−/−^ burned mice and controls. **j** Quantitative RT-PCR analysis of browning gene UCP1 in inguinal WAT of WT and UCP-1^−/−^ burned mice and controls. **k** Plasma concentration of free fatty acids in UCP-1^−/−^ burned mice and controls. **l** Oil Red O staining for fat droplets in liver sections from WT and UCP-1^−/−^ burned mice and controls. **m** Triglyceride (TG) content of livers from WT and UCP-1^−/−^ burned mice and controls. Data represented as mean ± SEM, *p* < 0.05 *significant difference WT burn vs. controls, *p* < 0.05 **#** WT burn vs. IL-6^−/−^ **/** UCP-1^−/−^ (*n* = 7, biological replicates, experiments repeated two times).

Recently, we have uncovered the cytokine interleukin 6 (IL-6) and type 2 macrophages in mediating catecholamine-induced UCP-1 expression and WAT browning during a burn injury^3,6,13^. To directy link WAT browning to the development of hepatic steatosis after a burn injury, we sought to block the two main regulators, IL-6 and UCP-1, involved in post-burn WAT browning. We first used IL-6 whole body KO (IL-6^−/−^) mice, in which mice lack the complete production of systemic IL-6, an upstream regulator previously implicated in both burn and cancer-induced WAT browning (Supplementary Fig. 1a–c). As expected, burn-induced weight loss and adipose tissue wasting was significantly attenuated in IL-6^−/−^ mice subjected to a burn injury (Fig. 2a, b). IL-6^−/−^ mice were also protected against burn-induced browning as genomic and histological analysis revealed diminished multilocular, UCP1+ adipocytes in the adipose post burn injury (Fig. 2c, d). Additionally, these IL-6^−/−^ mice did not show a significant increase in lipolysis compared to post-burn WT mice (Fig. 2e). In accordance with the above observations made in IL-6^−/−^, inhibition of WAT browning significantly decreased hepatic fat accumulation in these mice post-burn injury. Interestingly, liver weights of IL-6^−/−^ were lower compared to WT mice post-burn injury, indicating reductions in lipid infiltration (Fig. 2f). In agreement with our liver weight findings, hepatic lipid infiltration and liver TG content were all reduced in IL-6^−/−^ mice, compared to WT controls post-burn inury (Fig. 2g, h).

Furthermore, the browning gene UCP-1 has also been implicated as the downstream regulator of both cold and burn-induced WAT browning^14^. To further implicate WAT browning in post-burn hepatic steatosis, we next utilized UCP-1 KO (UCP-1^−/−^) mice in which the downstream regulator of post-burn WAT browning, namely, UCP-1 is not expresed. Histological and genomic analysis confirmed that UCP1 expression in WAT was completely ablated in UCP-1^−/−^ mice (Fig. 2i, j). Notably, loss of the UCP-1 gene in mice also attenuated burn-induced lipolysis, reduced hepatic lipid infiltration, and liver TG content (Fig. 2k–m). Together, these findings provide compelling evidence that inhibition of burn-induced browning via the deletion of either the upstream or downstream regulators, IL-6 and UCP-1, respectively, attenuates burn-induced hepatic steatosis.

### IL-6 receptor blockade and propranolol attenuates WAT browning and hepatic steatosis

**Fig. 3:**
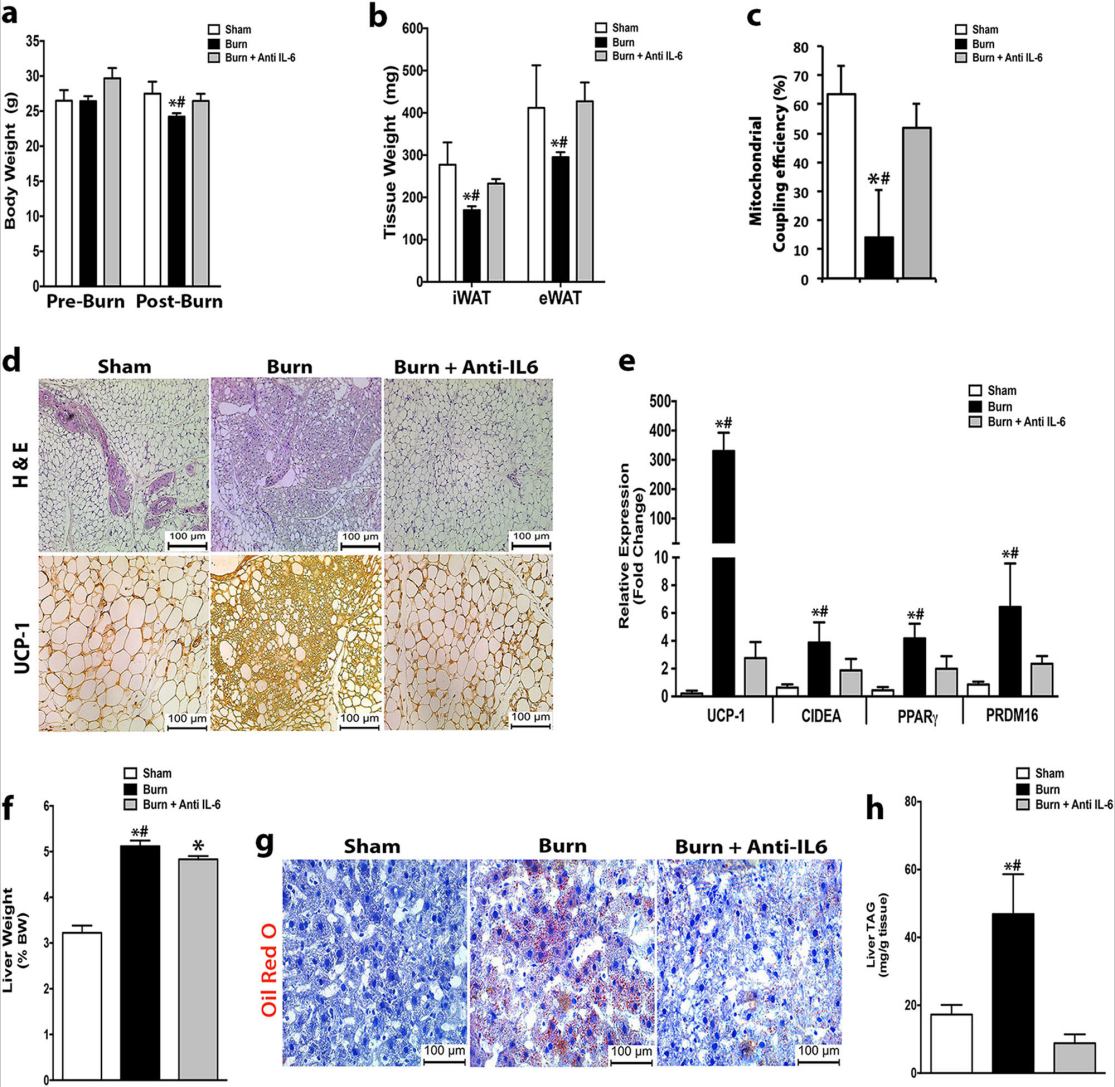
Blockage of IL-6 signaling post burn injury attenuates burn-induced WAT browning and hepatic steatosis. **a**, **b** Changes in total body (**a**) and fat (**b**) weight in post burn mice treated with vehicle or anti-mouse IL-6R monoclonal antibody daily for 5 days. **c** Mitochondrial coupling efficiency in inguinal WAT isolated from post burn mice treated with vehicle or anti-mouse IL-6R monoclonal antibody. **d** H&E and uncoupling protein 1 (UCP1) staining in inguinal WAT of post burn mice treated with vehicle or anti-mouse IL-6R monoclonal antibody. **e** Quantitative RT-PCR analysis of browning genes in inguinal WAT of post burn mice treated with vehicle or anti-mouse IL-6R monoclonal antibody. **f** Liver weights normalized to body weight of post burn mice treated with vehicle or anti-mouse IL-6R monoclonal antibody. **g** Oil Red O staining for fat droplets in liver sections from post burn mice treated with vehicle or anti-mouse IL-6R monoclonal antibody. **h** Triglyceride (TG) content of livers from post burn mice treated vehicle or anti-mouse IL-6R monoclonal antibody. Data represented as mean ± SEM, *p* < 0.05 *significant difference WT burn vs. littermate controls, *p* < 0.05 **#** WT burn vs. IL-6^−/−^ burn (*n* = 7, biological replicates, experiments repeated two times).

Thus far having shown that removing genes (IL-6, UCP-1) involved in post-burn WAT browning protected mice from hepatic steatosis, we wanted to determine if we could attenuate this adverse post-burn response therapeutically in wild type mice. To accomplish this, we tested the therapeutic potential of Tocilizumab, an IL-6 receptor (IL-6R) blocker that inhibits IL-6 signaling implicated in both burn and cancer-induced WAT browning^3,6,13^. Remarkably, administering the IL-6R blocker in post-burned mice significantly diminished the development of burn-induced hypermetabolism (weight loss, fat wasting) and recovered mitochondrial coupling (Fig. 3a–c). Consistent with reduced hypermetabolism, mice treated with the IL-6R blocker showed reduced UCP1+ cells and decreased gene expression of key browning markers (Fig. 3d, e). The therapeutic effects of IL-6R blocker treatment in the adipose tissue of post-burn mice also carried over to the liver, where we observed reductions in markers of hepatic steatosis (Fig. 3f–h). Similarly, we also examined the potential therapeutic effect of inhibiting WAT browning in post-burn mice via the catecholamine antagonist propranolol, an FDA approved drug currently in use in trauma and burn patients for cardiac purposes^15,16^. Interestingly, treatment of propranolol in post-burned mice significantly attenuated burn-induced hypermetabolism, WAT browning, and hepatic steatosis (Supplementary Fig. 2a–h). Taken together these findings provide further compelling evidence that WAT browning mediates post-burn hepatic steatosis and that blocking this process could serve as a potential therapeutic strategy to counteract its adverse effects on the liver.

### Elevated expression of ER stress inducing lipids suppress hepatic fat oxidation after a burn injury

**Fig. 4:**
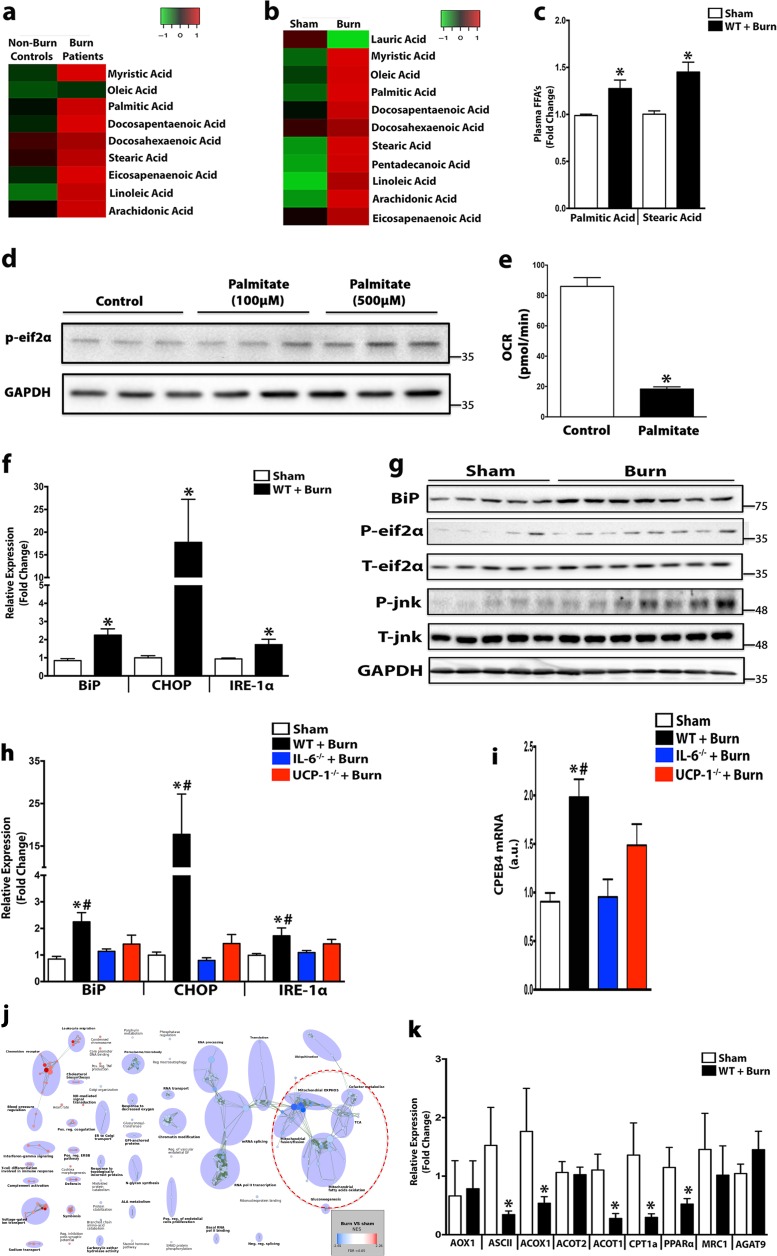
Lipodomic profiling after a burn injury reveals upregulation of ER stress inducing lipids. **a**, **b** Heat map display of lipid species within the plasma taken from burn patients and post-burn mice. **c** Quantification of FFAs (Palimitic and Stearic) in the plasma of wild type burned mice and controls. **d** Immunoblot of ER stress marker p-eif2a in HepG2 cells exposed to vehicle or Palimitate (100 μm or 500 m) for 24 h. **e** Oxygen Consumption Rate (OCR) in HepG2 cells exposed to vehicle or Palimitate (500μm) for 24 h. **f** Quantitative RT-PCR analysis of ER stress/UPR gene expression from livers of wild type (WT) burned mice and controls. **g** Immunoblot analysis of ER stress/UPR proteins in liver samples of wild type (WT) burned mice and controls. **h**, **i** Quantitative RT-PCR analysis ER stress/UPR and CPEB4 gene expression from the livers of UCP-1^−/−^ and IL-6^−/−^ burned mice and controls. **j** Parametric analysis of gene-set enrichment (PAGE) of the most highly upregulated (red) and down-regulated (blue) mitochondrial genes in livers of wild type (WT) burned mice and controls. **k** Quantitative RT-PCR analysis of beta-oxidation genes in the livers of wild type (WT) burned mice and controls. Data represented as mean ± SEM, *p* < 0.05 *significant difference WT burn vs. controls (*n* = 8, biological replicates, experiments repeated two times).

Having established hepatic steatosis occurs following burn-induced browning and lipolysis, we next focused on identifying and assessing the impact of the released lipids from the adipose tissue on hepatic function and fat oxidation. Lipid profiling in samples taken from both burn patients and post-burn mice using chromatography-mass spectrometry (LC-MS)-based global lipidomic analysis, revealed changes to a total of 9 (burn patients) and 11 (mice) circulating fatty acids in response to the injury (Fig. 4a, b). It has been reported that the accumulation of specific lipids (palmitic and stearic acid) is damaging in regards to hepatocyte function and survival, as these lipids lead to the activation of the unfolded protein response (UPR)^17^. We found a ~1.5 fold increase in the plasma expression of palmitic and stearic acid after a burn injury (Fig. 4c), which led us to postulate that ER stress may be at play in the context of browning-induced hepatic dysfunction. Indeed, treatment of in vitro hepatocytes with palimitate resulted in ER stress activation and decreased mitochondrial respiration (Fig. 4d–e). Similarly, in livers from mice that have undergone WAT browning and developed hepatic steatosis after a burn injury, we saw increased gene and protein expression of ER stress markers BiP, CHOP, IRE-1α, and phosphorylated forms of JNK and eIF2α (Fig. 4f, g). Conversely, both UCP1^−/−^ and IL-6^−/−^ mice that were protected from both burn-induced WAT browning and hepatic steatosis, also showed reductions in hepatic ER stress (Fig. 4h). Additionally, the cytoplasmic polyadenylation element-binding (CPEB-4) protein, which is only synthesized following ER stress, was upregulated in post-burn mice and attenuated in post-burn mice where WAT browning was inhibited (Fig. 4i).

Furthermore, studies have also reported that hepatic ER stress can exacerbate organ steatosis by inhibiting genes involved in lipid catabolism^18,19^. To directly assess this, we performed microarray analysis in livers from burned mice, which showed a substantial reduction in genes involved in oxidative metabolism and lipid transport (Fig. 4h). Confirming our microarray data, expression of genes associated with β-oxidation such as proliferator-activated receptor alpha (Pparα), carnitine palmitoyltransferase 1α (Cpt1α), and peroxisomal and microsomal oxidation genes (Acox1, Aox1) were also suppressed in the livers of post-burn mice (Fig. 4i). These findings demonstrate that burn-induced WAT browning and the associated release of lipids not only augments the development of hepatic steatosis, but also impairs the ability of the liver to mitigate the infiltration of these lipids by suppressing fatty acid oxidation.

### Hepatic ER stress facilitates increased ER-mitochondrial coupling after a burn injury

Having identified reduced hepatic fat oxidation after a burn injury, we next sought to uncover how hepatic ER stress mediated this response at the organelle level. Recently, it has been reported that hepatic lipid transport and metabolism have been shown to be enriched in contact membranes between the ER and mitochondria termed the mitochondria-associated membrane (MAM), and that hepatic ER stress facilitates increased MAM formation^20–22^.

**Fig. 5:**
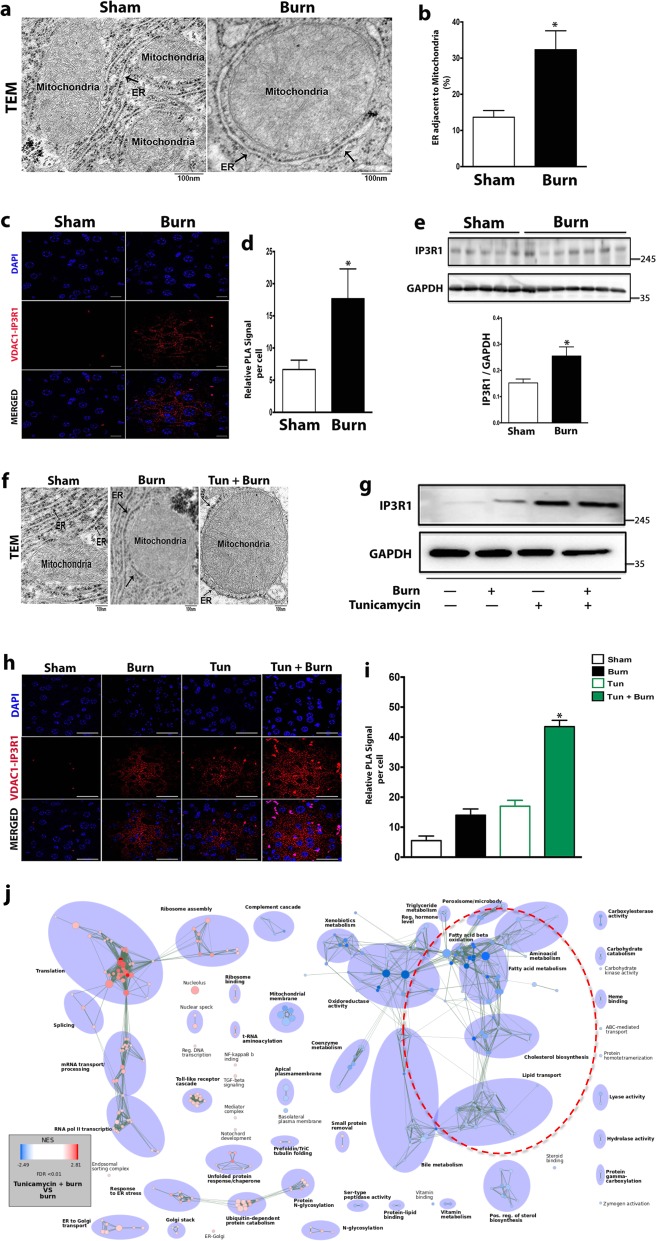
Hepatic ER stress post-burn injury leads to increased ER-Mitochondrial interaction. **a**, **b** Representative TEM images and quantification (scale bare = 100 nm) illustrating ER-Mitochondria tethering in liver sections derived from wild type (WT) burned mice and controls. **c**, **d** Representative PLA images (×63 magnification) and quantification of VDAC1/IP_3_R1 interactions (small red dots around the nucleus) in livers from wild type (WT) burned mice and controls. **e** Representative western blot and quantification of MAM enriched protein IP_3_R1 in livers of wild type (WT) burned mice and controls. **f** Representative TEM images (scale bare = 100 nm) illustrating ER-mitochondria tethering of liver sections derived Sham, Burn, and Tun + Burn mice. **g** Representative western blot of MAM enriched protein IP3R1 in livers of Sham, Burn, Tun., and Tun + Burn mice. **h**, **i** Representative PLA images (×63 magnification) and quantification of VDAC1/IP_3_R1 interactions (small red dots around the nucleus) in livers of Sham, Burn, Tun., and Tun + Burn mice. **j** Parametric analysis of gene-set enrichment (PAGE) of the most highly upregulated (red) and down-regulated (blue) mitochondrial genes in livers of burned mice and Tun + Burn mice. Data represented as mean ± SEM, *p* < 0.05 *significant difference WT burn vs. control, *p* < 0.05 **#** WT burn vs. Tun + Burn (*n* = 8, biological replicates, experiments repeated two times).

Indeed, we not only found the deregulation of genes involved in maintaining normal MAM formation^22,23^ (Supplementary Fig. 3a, b), but we also observed a higher degree of ER tethering to the mitochondria in mice subjected to a burn (Fig. 5a, b). To further confirm increased hepatic MAM enrichment and ER-mitochondria coupling, we employed an organelle targeted in situ proximity ligation assay (PLA). Using in situ PLA by targeting the ER organelle surface marker inositol 1,4,5-trisphosphate receptor (IP_3_R) and mitochondrial surface marker voltage dependent anion channel (VDAC), we were able to quantify the level of interaction between the two organelles. Confirming our TEM observations, we found significantly increased ER-mitochondria cross-talk in liver sections from burned mice (Fig. 5c, d). Additionally, we found an increased expression of the MAM enriched protein IP_3_R1 in liver lysates of burned mice (Fig. 5e).

To confirm the changes in hepatic ER-Mitochondrial coupling resulted from hepatic ER stress as has previously been reported^22^, we examined the effect of acute ER stress in the liver in vivo by administration of the ER stress-inducing drug tunicamycin as a “second hit” in post-burn mice. Tunicamycin treatment in post-burn mice resulted in a higher degree of ER-mitochondria interaction and increased expression of the MAM enriched protein IP3R1 than what we observed earlier in burned mice alone (Fig. 5f, g). The greater degree of ER-mitochondrial interaction observed in burn mice treated with tunicamycin was corroborated by in situ proximity ligation assay (Fig. 5h, i). In parallel, we also performed genome-wide microarray analysis in the livers of burn mice treat with tunicamycin, which showed greater suppression of genes involved in β-oxidation compared to burned mice alone (Fig. 5j). These ER-stress induced alterations at the organelle level also resulted in hepatic dysfunction as markers of hepatic damage such as alanine aminotransferase (ALT), hepatocyte proliferation (as determined by Ki-67 staining), and apoptosis, as determined by TUNEL staining and caspase 3 activation, were significantly upregulated in both post-burn mice and burn mice treated with tunicamycin (Supplementary Fig. 4a–d). Collectively, our findings indicate that hepatic ER stress facilitates alterations at the organelle level between the ER and mitochondria in order to suppress fat oxidation.

### Cell-permeant IP_3_R peptide potently blocks ER stress induced hepatocyte apoptosis

**Fig. 6:**
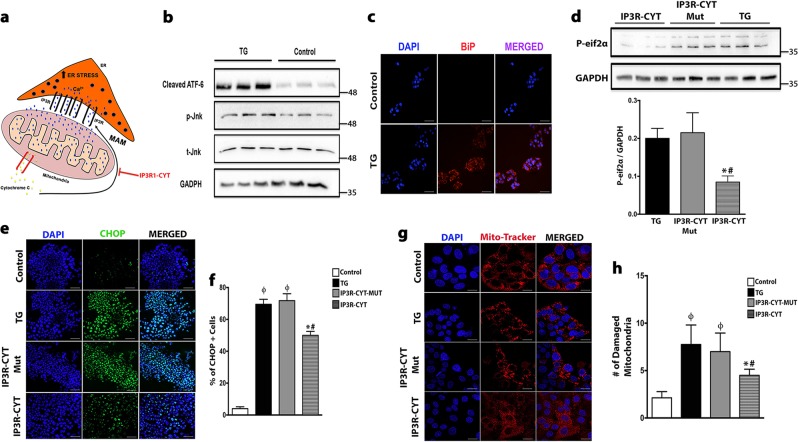
A cell permeant IP_3_R peptide attenuates ER stress and blocks apoptosis in vitro. **a** A schematic illustrating the consequences of increased hepatic ER stress and MAM enrichment that can lead to an efflux of excessive Ca^2+^ from the ER via the IP_3_R receptor to the mitochondria. The by-product of mitochondrial damage (cytochrome c) feedbacks to the IP_3_R to sustain this cycle that ultimately results in cellular apoptosis. **b** Immunoblot of ER stress proteins c-ATF6 and p-jnk in HepG2 cells treated with either TG (100 nM) for 24 h or vehicle. **c** Immunofluorescence staining of ER stress marker BiP (red) in HepG2 cells treated with either TG (100 nM) for 24 h or vehicle. **d** Immunoblot and quantification of ER stress protein p-eif2α from HepG2 cells treated with either TG (100 nM) for 24 h, TG (100 nM) treated cells incubated with either BODIPY-IP_3_RCYT-MUT(400 nM), and or BODIPY-IP_3_R-CYT(400 nM) for 4 h. **e** Immunofluorescence staining of pro-apoptotic marker CHOP (green) in HepG2 cells treated with either TG (100 nM) for 24 h, TG (100 nM) treated cells incubated with either BODIPY-IP_3_RCYT-MUT(400 nM), and or BODIPY-IP_3_RCYT(400 nM) for 4 h. **f** Quantification of Immunofluorescence staining of CHOP from (**e**). **g** Immunofluorescence staining of mitochondria with Deep Red MitoTracker (red) in HepG2 cells treated with either TG (100 nM) for 24 h, TG (100 nM) treated cells incubated with either BODIPY-IP_3_RCYT-MUT(400 nM), and or BODIPY-IP_3_RCYT(400 nM) for 4 h. **h** Quantification of Immunofluorescence staining of the MitoTracker from (**g**). Data represented as mean ± SEM, *p < 0.05 vs. control or TG, ^ϕ^*p* < 0.05 vs. control ^#^*p* < 0.05 vs. IP3RCYT-MUT/ TG (*n* = 7, biological replicates, experiments repeated two times). Scale bars represent ×20 magnification for (**c**, **e**), and ×60 magnification for (**g**).

Hepatic ER stress and increased MAM enrichment have also been implicated in mediating hepatocyte apoptosis. Indeed, others and we have previously reported that increased hepatic ER stress and IP_3_R1 facilitates increased calcium shuttling from the ER to the mitochondria to induce cellular apoptosis^22^. During cellular apoptosis, it has also been reported that Cytochrome C exits the mitochondria and binds directly and selectively to IP_3_R1 in the closely adjacent ER, thereby amplifying calcium-dependent apoptosis^24,25^. Using the drug thapsigargin, which facilitates ER-calcium depletion and ER stress, we aimed to study the therapeutic effect of a cell permeable peptide (IP_3_R-CYT) that displaces Cytochrome C from IP_3_R, thereby blocking calcium-induced apoptosis and ER stress (Fig. 6a–c). We were able to abrogate key ER stress markers, specifically phosphorylated eIF2α and CHOP, induced by thapsigargin with the use of the IP_3_R peptide that displaces Cytochrome C from IP_3_R (Fig. 6b, c, e). Treating these HEPG2 cells with the IP_3_R peptide also attenuated ER stress induced mitochondrial damage (Fig. 6d, f). Thus, our data demonstrate that directly targeting proteins that mediate ER-mitochondria cross talk might be a promising therapeutic strategy to mitigate the adverse cellular effects of hepatic ER stress after a burn injury.

## Discussion

**Fig. 7:**
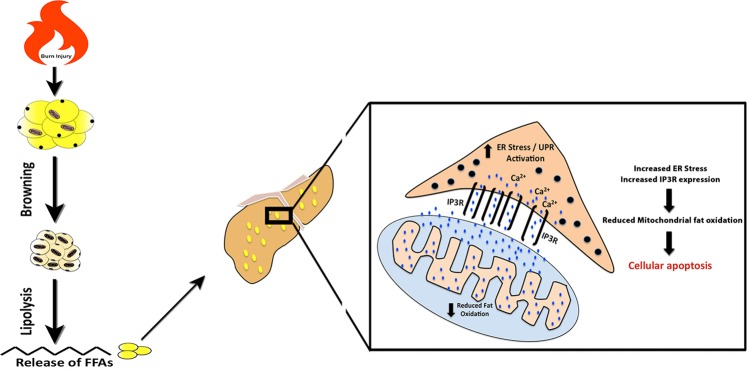
Schematic diagram illustrating the consequences of WAT browning and its associated lipotoxicity for the liver after a burn injury. Following a burn injury you have the activation of the browning process whereby the white adipose tissue converts to beige fat. Pro-inflammatory FFAs released from beige fat than travel to the liver causing hepatic steatosis and hepatic dysfunction by activating the ER stress response in hepatocytes. Hepatic ER stress then leads to increased expression of the MAM enriched protein IP_3_R1, which leads to a greater ER-Mitochondria interaction and facilitates Ca^2+^ transfer from ER (via IP_3_R1) to the mitochondria. This, in turn, can lead to reductions in mitochondrial fat oxidation. If this chronic ER stress mediated by lipid infiltration is not mitigated it can ultimately result in cellular apoptosis.

Current understanding of the activation of WAT browning in hypermetabolic conditions (burns, cancer) is poorly understood, and whether such browning has deleterious effects on outcome and organ function are even less known. In this study, we provide evidence to support the adverse effects of beige fat in a burn injury model of hypermetabolism. We provide evidence that browning of WAT after a burn injury triggers lipolysis, which in turn leads to an increased blood lipid profile that culminates in hepatic steatosis and dysfunction via ER stress (Fig. 7). This effect appeared to be browning mediated as blockage of WAT browning via the upstream regulator (IL-6) and the downstream regulator (UCP-1) in mice attenuated hepatic steatosis after a burn injury. We also identified the pathogenesis of the infiltrated lipids for the liver, as lipidomic profiling of the plasma of both burned mice and patients revealed the expression of palmitic and stearic acid, which mediated hepatic ER stress and the suppression of fat oxidation. Mechanistically, we also uncovered abnormal ER-mitochondria cross talk via MAM enrichment and IP_3_R1 expression as the cellular mechanisms behind lipid-induced hepatic dysfunction and apoptosis.

To date, WAT browning has been described as a beneficial event in the context of obesity and diabetes, as it has been shown to promote weight loss and improve insulin sensitivity in both metabolic conditions^26,27^. However, browning in the context of cancer has recently been shown to be detrimental, as it facilitates cancer-induced wasting and cachexia^3,5^. Burn injury, which induces a similar hypermetabolic state as seen in cancer, was also recently shown to induce browning, although whether such browning is actually detrimental in the burn context is still unknown. Here, we show that burn injury induced browning mediates hepatic steatosis and adverse alterations at the cellular level. The liver is a focal point for lipid mobilization that oxidizes lipids for glucose during fasting and packages these lipids into lipoproteins for peripheral tissues^28,29^. In the lipid-accumulated livers of post burn mice, we observed the activation of ER stress, which was non-existent in burned mice where browning was inhibited. It has been previously reported in studies outside of burns that ER stress leads to lipid accumulation and hepatic steatosis via the inhibition of beta-oxidation^18,30^. Indeed, our findings in the livers of post-burn mice corroborate these past findings and indicate that lipid-induced hepatic ER stress impairs fat oxidation, which in turn further facilitates fat accumulation in this organ.

Our study also aimed to develop a deeper understanding of the cellular changes that take place after a burn injury in the hopes of resolving how browning-induced hepatic steatosis and ER stress leads to suppression of hepatic fat oxidation. Although the precise mechanisms of how ER stress fosters the suppression of fat oxidation remains elusive, it likely involves the physical interaction between the ER and mitochondria, namely the MAM. The MAM not only establishes a physical and functional connection between the ER and the mitochondria, but also regulates fat metabolism and calcium signaling^31^. The attendant crosstalk between the ER and mitochondria appears to be vital for cell integrity, fat metabolism, death, and maintenance of function^32^. In fact, it has recently been shown that abnormal communication between the ER and mitochondria in obesity has damaging effects on mitochondrial and cellular function^33^. Our findings in which after a burn injury hepatic ER stress results in greater ER-mitochondrial interaction and the expression of the MAM enriched IP_3_R protein support these findings albeit in the context of a response to a traumatic injury. It has also been reported that calcium depletion from the ER via the IP_3_R receptor leads to calcium overload in the mitochondria, which in turn activates a feed-forward cascade whereby released cytochrome c interacts with the IP_3_R receptor to further amplify calcium release and eventually lead to hepatocyte apoptosis^24,25^. The hepatocyte apoptosis after a burn injury we observed likely resulted from ER stress induced IP_3_R1 over expression, as our in vitro findings showed that hepatocyte ER stress and mitochondrial dysfunction were both abrogated by a cell permeant peptide that displaces cytochrome c from IP_3_R. Therefore, we suggest that targeting proteins that regulate the ER-mitochondrial interface in order to restore normal function, maybe a useful approach to mitigate the effects of lipid-induced hepatocyte ER stress and cell death.

Our findings also highlighted the potential therapeutic benefits of blocking the browning process after a burn injury for the liver. Based on our previous findings in which we uncovered IL-6 and type 2 macrophages as the regulators of post-burn browning, we envisioned that blocking IL-6 signaling via the use of an IL-6 receptor-neutralizing (IL-6R) antibody might be therapeutically beneficial in alleviating both WAT browning and hepatic steatosis. Indeed, blocking the IL-6R was able to inhibit adipose browning and its associated hepatic steatosis in post-burned mice. These findings are critical because most of the work in blocking WAT browning has focused on blocking the β-adrenergic receptors, leaving other potential avenues relatively unexplored. Interestingly, it has been reported that patients receiving the IL-6R neutralizing mAb Actemra (tocilizumab) for rheumatoid arthritis show side-effects like an increase in body weight and marked hypercholesterolemia during the treatment period, which maybe a result of an inhibition of WAT browning in these patients who also have chronic IL-6 production. Thus, while our study broadens the understanding of WAT browning in contexts where it plays a detrimental rather than a beneficial role, further studies are warranted to examine the effects of browning in other tissues outside the liver.

## Materials and methods

### Human samples

Patients admitted to the Ross Tilley Burn Centre at Sunnybrook Hospital (Toronto, Canada) or non-burn patients undergoing elective surgery were consented pre-operatively for blood collection. Approval for our study was obtained from the Research Ethics Board at Sunnybrook Hospital.

### Animals

Male C57BL/6 and IL6^−/−^ mice (Jackson, USA) were housed at thermoneutral temperature (28 °C) and cared in accordance with the Guide for the Care and Use of Laboratory Animals. Congenic lines of UCP-1^−/−^ mice were generated by backcross matings of heterozygous (+/–) mice on a mixed 129/SvPas and C57BL/6J background with 129/SvImJ and C57BL/6J mice as previously described (Jackson, USA). In this study, all wild-type mice are C57BL/6J and are referred to as wild type (WT) and all UCP-1 mutant mice are on the C57BL/6J background and are referred to as UCP1^−/−^. Randomization of animals was done by the animal tech that divided the animal cohorts into groups of 5 in a cage after arrival to the facility. These cohorts were then treated as described in the paper. Mice were housed and cared in accordance with the Guide for the Care and Use of Laboratory Animals and procedures approved by the Sunnybrook Research Institute Animal Care Committee under AUP 467 (Toronto, Ontario, Canada). Due to the nature of the burn injury, size estimates and the number animals per group were done in consultation with the Animal Care Committee at Sunnybrook Research Institute. All experiments were performed with 2 month old male WT and UCP1^−/−^ mice. Mice receiving an injury were subjected to a 30% total body surface area (TBSA) full thickness burn. All mice were subsequently housed individually in sterile cages and fed ad libitum until sacrifice. Tissues were harvested 5 days post burn injury and used for analysis.

### Mouse burn injury model

Mice were anaesthetized using ketamine (0.15 mg/g) and xylazine (0.01 mg/g). The dorsum of the trunk was shaved and 1.5 ml Ringer’s Lactate was injected subcutaneously along the spine and 0.5 ml Ringer’s Lactate was injected intraperitoneally. Mice were placed in a mold that exposed 30% total body surface area of the shaved dorsum. A full thickness cutaneous scald burn was administered by lowering the mold into a 98 °C water bath for 10 s. Mice in the sham group were shaved, anaesthetized and received Ringer’s Lactate.

### Histology and immunohistochemistry

Adipose tissue was immediately fixed in 10% formalin and then maintained in 70% ethanol prior to paraffin embedding. Subsequently, tissues were sectioned and stained with Haematoxylin and Eosin (H&E) or incubated with UCP1 (Sigma) antibody followed by DAB staining. Livers from mice were isolated and immediately frozen in a tissue freezing medium (O.C.T. compound, Tissue-Tek) and stored at −80 °C until staining. Imaging was performed on a LSM confocal microscope (Zeiss, Germany). The investigator was blinded after sample collection for purposes of histology imaging and analysis.

### Quantitative PCR

Total RNA isolated from adipose tissue, and primary macrophages was analyzed by quantitative RT-PCR. RNA was isolated from tissue and cells using TRIzol-chloroform (Life Technologies) with subsequent purification using the RNeasy Kit (Qiagen) according to the manufacturer’s instructions. RNA (2 mg) was transcribed to cDNA using the high capacity cDNA reverse transcription kit (Applied Biosystems). Real-time quantitative PCR was performed using the Applied Biosystems Step One Plus Real-Time PCR System. Primer sequences used are available upon request.

### Microarray analysis

The liver was dissected from each mouse and homogenized, total RNA was extracted using a QIAGEN kit according to the manufacturer’s instructions. For gene profile analysis, RNA quality was assessed with a Bioanalyzer (Agilent Technologies), and samples with an RNA purity greater than 1.8 were included for array. cDNA was generated and hybridized onto the Affymetrix Mouse Gene 1.0 ST chips. Analysis of gene expression was performed using Parktec Genotyping Suite and Ingenuity Systems Software.

### Statistical analysis

All data are presented as mean ± s.e.m and analyzed using Prism (Graphpad). Statistical significance was determined using a Student’s t-test or one-way ANOVA followed by a Bonferroni posthoc tests were used where appropriate. A *p*-value of <0.05 was considered to be statistically significant, and is presented as * (*p* < 0.05). Samples were excluded as outliers if they were 2 standard deviations above or below the mean. Tests were done that showed the variance was equal among the different groups.

Other Detailed experimental procedures and protocols are described in the Supplementary Material.

## Supplementary information

Extended Methods

Supplemental Figure 1

Supplemental Figure 2

Supplemental Figure 3

Supplemental Figure 4
