# Supplementary material for: Browning of white adipose tissue after a burn injury promotes hepatic steatosis and dysfunction
Source: Cell Death Dis. 2019 Nov 18;10(12):870. doi: 10.1038/s41419-019-2103-2 (PMC6861318; doi:10.1038/s41419-019-2103-2)
Supplement: Supplementary file 1 — Extended Methods [file 41419_2019_2103_MOESM1_ESM.docx]

**EXTENDED EXPERIMENTAL PROCEDURES AND SUPPLEMENTAL FIGURES**

**Western Blotting.** Fat and liver tissues were lysed in RIPA buffer (50mM Tris-HCl pH 7.5, 150mM NaCl, 1% Igepal, 0.5% sodium deoxycholate, 0.1% SDS, 1mM NaF and protease inhibitors) using tissue beater. Total protein (40 mg) was separated on SDS– PAGE gels, transferred to nitrocellulose membrane, and incubated with primary antibodies directed against UCP1 (Sigma), p-eif2a, CHOP, p-JNK (Cell Signaling), IP3R1(Santa Cruz), and α-β Tubulin (Cell Signaling), Proteins were visualized by enhanced chemiluminescence.

**GC/MS:** Serum fatty acids from patients and mice were analyzed by gas chromatography–mass spectrometry (GC–MS) performed by the Analytical Facility for Bioactive Molecules (AFBM) platform at the Hospital for Sick Children, Toronto, Ontario, Canada. Briefly, serum samples (20 µL) were spiked with an internal standard mix and acidified with HCl. Non-esterified fatty acids were acidified and double-extracted with hexane. The fatty acids were then converted to their pentafluorobenzyl esters using 1% pentafluorobenzyl bromide/diisopropylamine (1:1) and separated by automated gas chromatography (GC Agilent 7890A, Agilent Technologies, Santa Clara, CA, USA) on a fused-silica SP2380 capillary column (30 m × 0.25 mm x 0.2 µm film thickness; Supelco Analytical, Bellefonte, PA, USA). Fatty acid ions were detected and measured using a MSD Agilent 5975C quadrupole mass detector (Agilent Technologies, Santa Clara, CA, US). Peaks of fatty acid esters were identified by comparisons with individual fatty acid standards (Supelco Analytical, Bellefonte, PA, USA). Individual fatty acid concentrations (ng/µL) were calculated from the area peak of the internal standards using the Agilent ChemStation software (Agilent Technologies, Santa Clara, CA, US). Saturated (SFA), monounsaturated (MuFA), or polyunsaturated (PuFA) fatty acid contents were calculated by combining the appropriate individual fatty acids into their respective classes (SFA- myrisitic/C14:0, palmitic/C16:0, and stearic acid/C18:0; MuFA- palmitoleic/C16:1 n-7, cis-7-hexadecenoic/C16:1 n-9, oleic/C18:1 n-9, vaccenic/C18:1 n-7, and eicosenoic/C20:1 n-7; PuFA- linoleic/C18:2, α-linolenic/C18:3, arachidonic/C20:4), eicosapentaenoic/C20:5, docosapentaenoic/C22:5), and docosahexaenoic/C22:6 acid).

**Respiration Assays:** Oxygen consumption rate (OCR) were measured in freshly-excised adipose tissues (inguinal adipose depots) were minced in mitochondrial isolation buffer (MHSE + BSA; 210 mM mannitol, 70 mM sucrose, 5mM HEPES, 1 mM EGTA, 0.5% (w/v) fatty acid-free BSA, pH 7.2). The tissue was then homogenized using a Teflon glass homogenizer. Mitochondria were isolated via differential centrifugation. Briefly, the homogenate was centrifuged at 600 g for 10 min and the supernatant decanted into a new tube. This fraction was centrifuged at 9000 g for 10 min to obtain a mitochondrial pellet, which was subsequently resuspended in 50 uL of MHSE + BSA. BCA assays (Thermo Scientific) were performed to gauge protein concentrations. Mitochondrial bioenergetics were assessed using a Seahorse XF96 analyzer (Agilent Technologies). Mitochondrial respiration in a coupled state (10 μg/well) was measured in mitochondrial assay solution (MAS; 220 mM mannitol, 70 mM sucrose, 10 mM KH2PO4, 5 mM MgCl2, 2 mM HEPES, 1 mM EGTA and 0.2% (w/v) fatty acid-free BSA, pH 7.2 at 37°C) containing succinate as a substrate (10 mM) and rotenone (2 μM). State 3 respiration (phosphorylating respiration) was triggered via the injection of a cocktail containing 4 mM ADP along with 10 mM pyruvate, 2.5 mM glutamate and 2.5 mM malate. State 4o respiration was assessed by the addition of 2.5 μg/mL oligomycin, while maximal uncoupler-stimulated respiration was observed following the injection of 4 μM carbonyl cyanide 4-(trifluoromethoxy)phenylhydrazone (FCCP). Antimycin A (4 μM), a complex III inhibitor, was added at the end of the experiment to inhibit mitochondrial respiration. The Seahorse XF Wave software was used to analyze the data.

**Transmission Electron Microscopy**. Inguinal fat pads and liver tissues were fixed in phosphate buffer containing 4% paraformaldehyde and 1% glutaraldehyde followed by post-fixation in phosphate buffer containing 1% osmium tetroxide. The tissues were dehydrated using a graded series of ethanol, washed with propylene oxide and infiltrated with Epon Araldite (E/A) resin using a graded series of E/A resin and propylene oxide. Following polymerization, tissue sections (70µm thick) were counterstained using saturated uranyl acetate followed by lead citrate and imaged using the Hitachi H7000 transmission electron microscope.

**Lipid measurements.** Triglycerides and free fatty acids (Biovision, Abcam) were quantified in duplicate as per the manufacturers’ protocols. For liver triglycerides, tissues were homogenized by sonification in homogenization buffer (0.01N HCl, 1 mM EDTA, 4 mM Na2S2O5) or manufacturer provided buffer, and cellular debris was pelleted by centrifugation at 13,000 rpm for 15 min at 4 C. The cleared homogenates were collected and stored in -80 C freezer prior to quantification.

**Induction of ER Stress by Tunicamycin.** Tunicamycin from Streptomyces sp. (Sigma) was dissolved in dimethyl sulfoxide (DMSO) and diluted in sterile 150mM dextrose to obtain a tunicamycin concentration of 10µg/µl. Mice (20-25g) were randomized into 3 groups, in which they either received a burn injury, or injected intraperitoneally with tunicamycin solution (1µg/g body mass) 24hrs pre-burn or 24hrs post burn and assessed for survival. As controls, mice were injected intraperitoneally with control buffer (150mM dextrose containing 1% DMSO).

**Cell Culture.** HepG2 cells were cultured in Dulbecco’s Modified Eagle Medium (DMEM, 4.5 g/L glucose) supplemented with 10% FBS and 1% antibiotics. 3T3L1 adipocytes were maintained in DMEM (4.5 g/L glucose) supplemented with bovine calf serum and differentiated for 7 to 10 days following incubation with a differentiation protocol previously described ([Wang et al., 1998](#_ENREF_40)). HepG2 cells were seeded in wells 24-48 hours prior to treatments, and treated 24hrs with either control buffer, thapsigargin (100nM) (Sigma Aldrich, St. Louis, MO, USA) or thapsigargin followed by IP3R-Mut or IP3R-Cyt peptide diluted in medium for an additional 4hrs.

**BODIPY-IP3R Peptide***.* Peptide synthesis and purification were performed at University of Texas Health Science Center in Houston. Coupling of the peptide to BODIPY 577/618 maleimide was performed as suggested by the manufacturer (Molecular Probes). The version of the peptide used in this manuscript was a gift from Dr. Darren Boehning at the University of Texas Health Science Center at Houston.
