# Supplementary figures and images for: Browning of white adipose tissue after a burn injury promotes hepatic steatosis and dysfunction
Source: Cell Death Dis. 2019 Nov 18;10(12):870. doi: 10.1038/s41419-019-2103-2 (PMC6861318; doi:10.1038/s41419-019-2103-2)

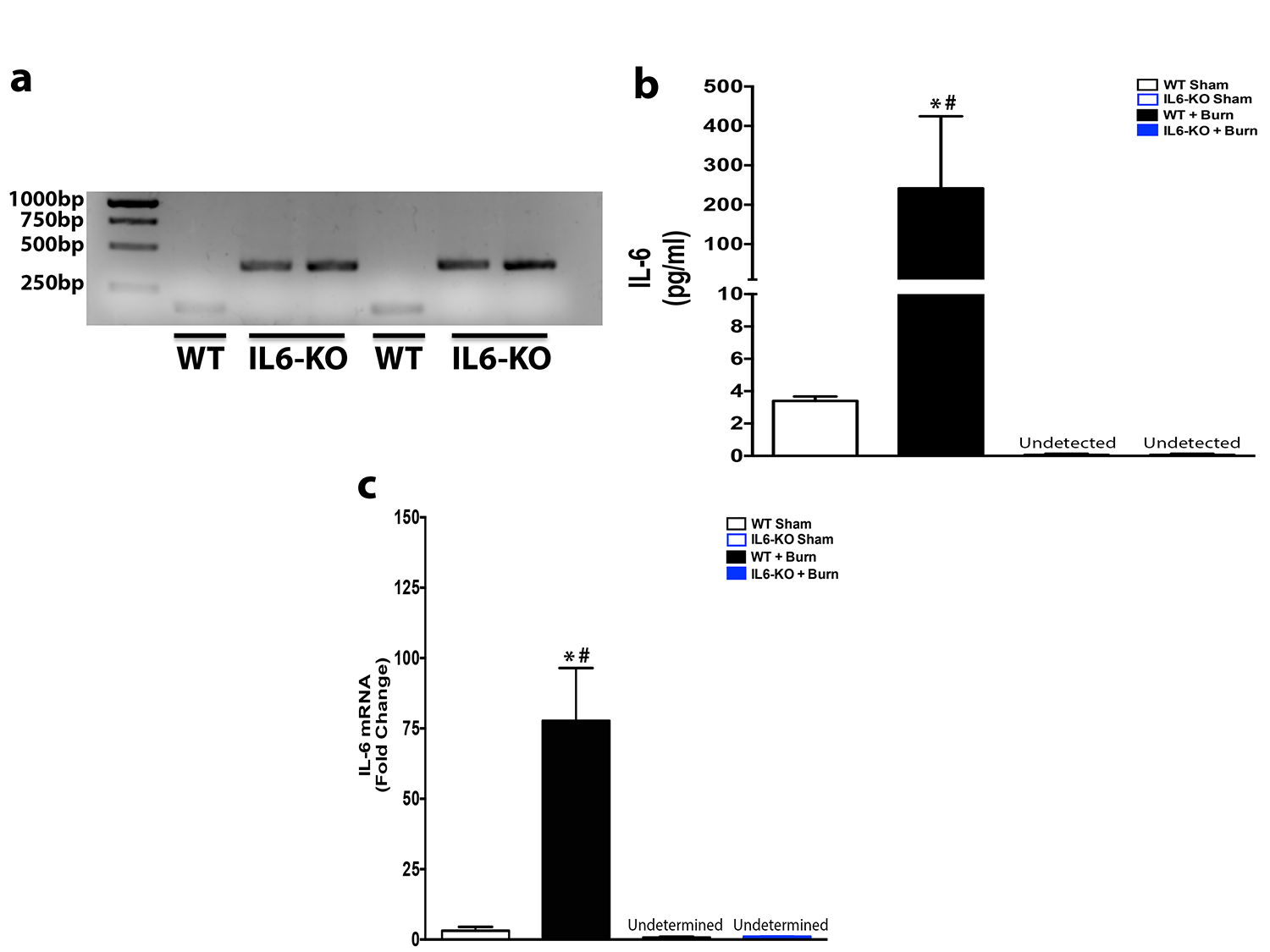

Supplement: Supplementary file 2 — Supplemental Figure 1 [file 41419_2019_2103_MOESM2_ESM.tif]

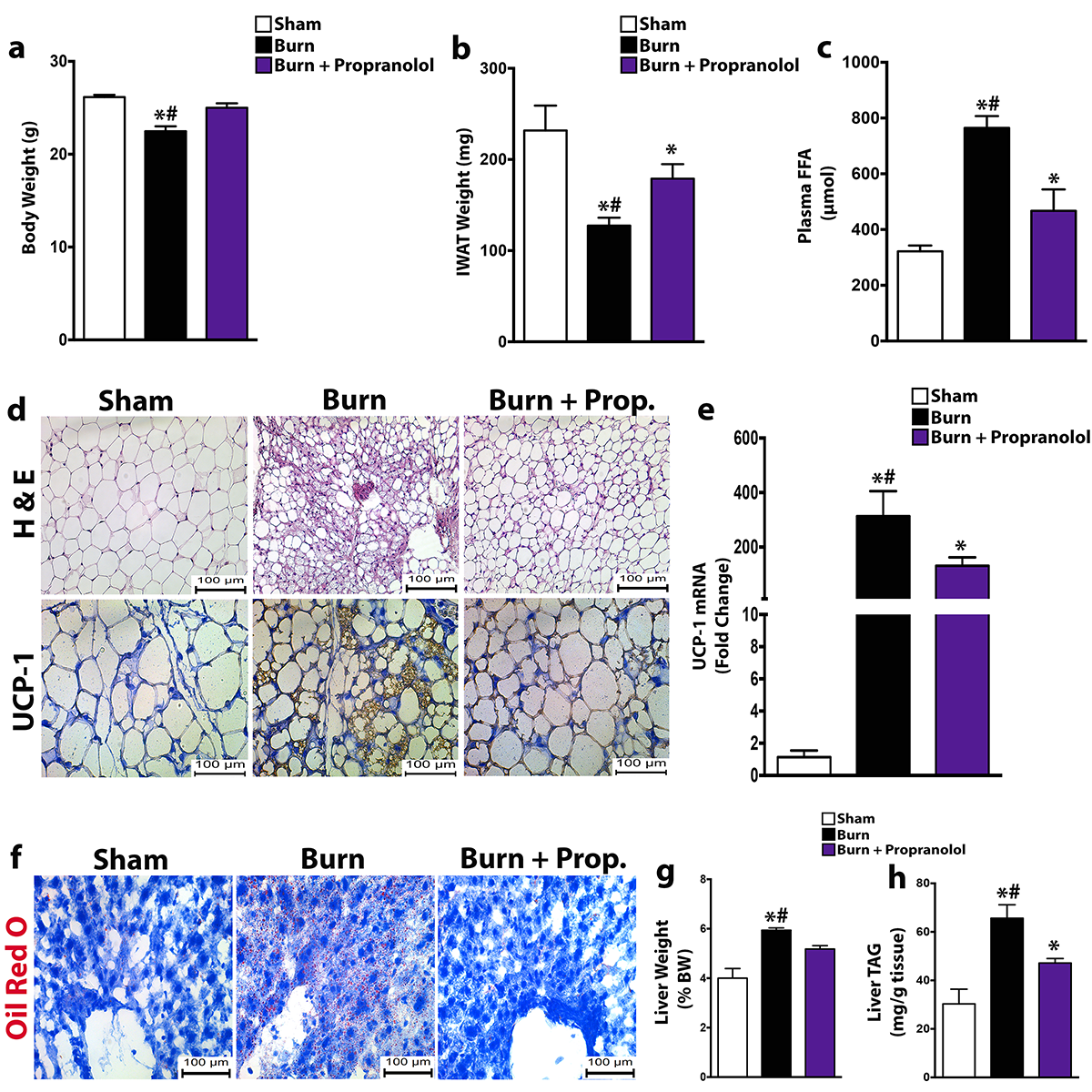

Supplement: Supplementary file 3 — Supplemental Figure 2 [file 41419_2019_2103_MOESM3_ESM.tif]

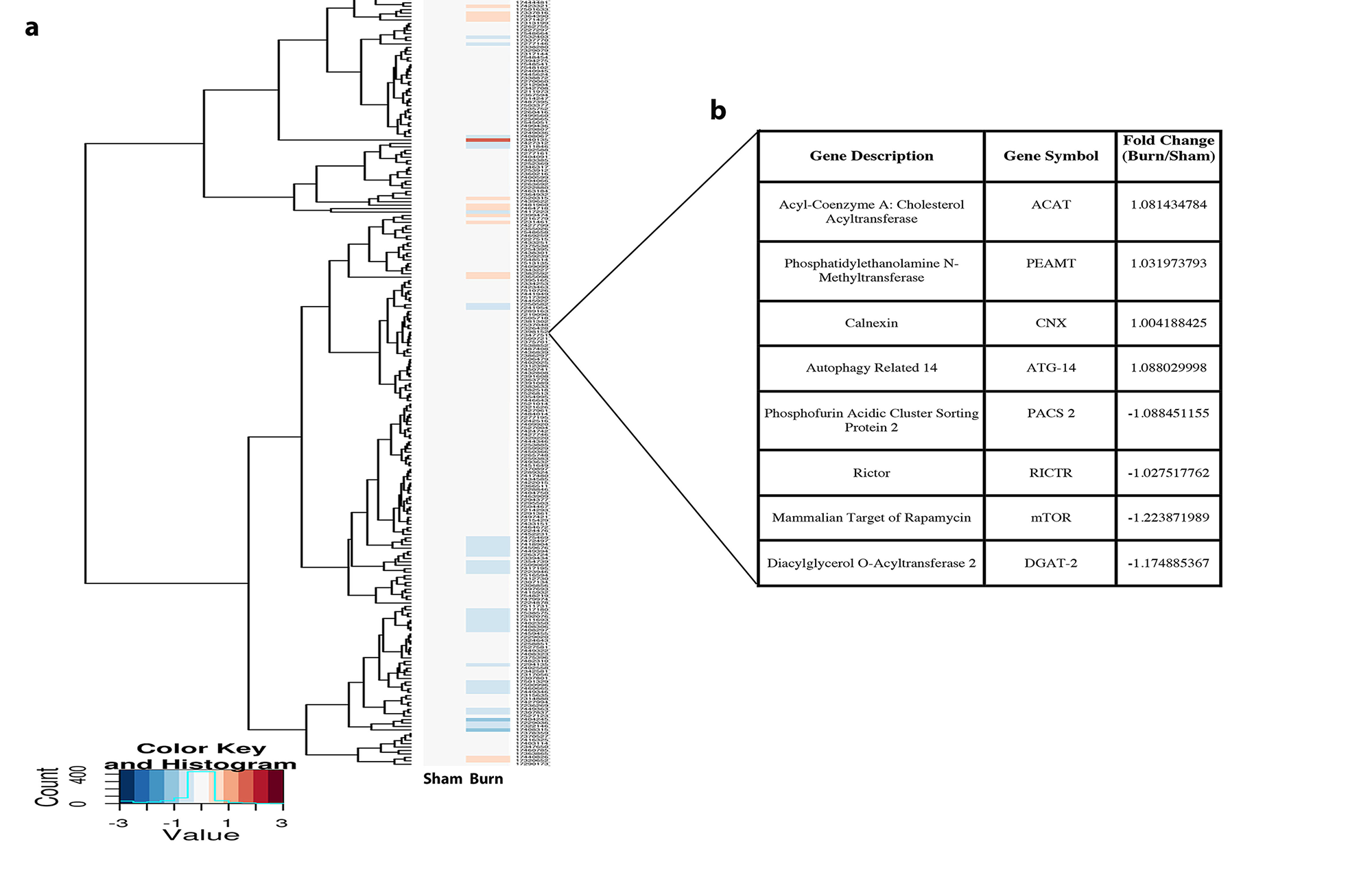

Supplement: Supplementary file 4 — Supplemental Figure 3 [file 41419_2019_2103_MOESM4_ESM.tif]

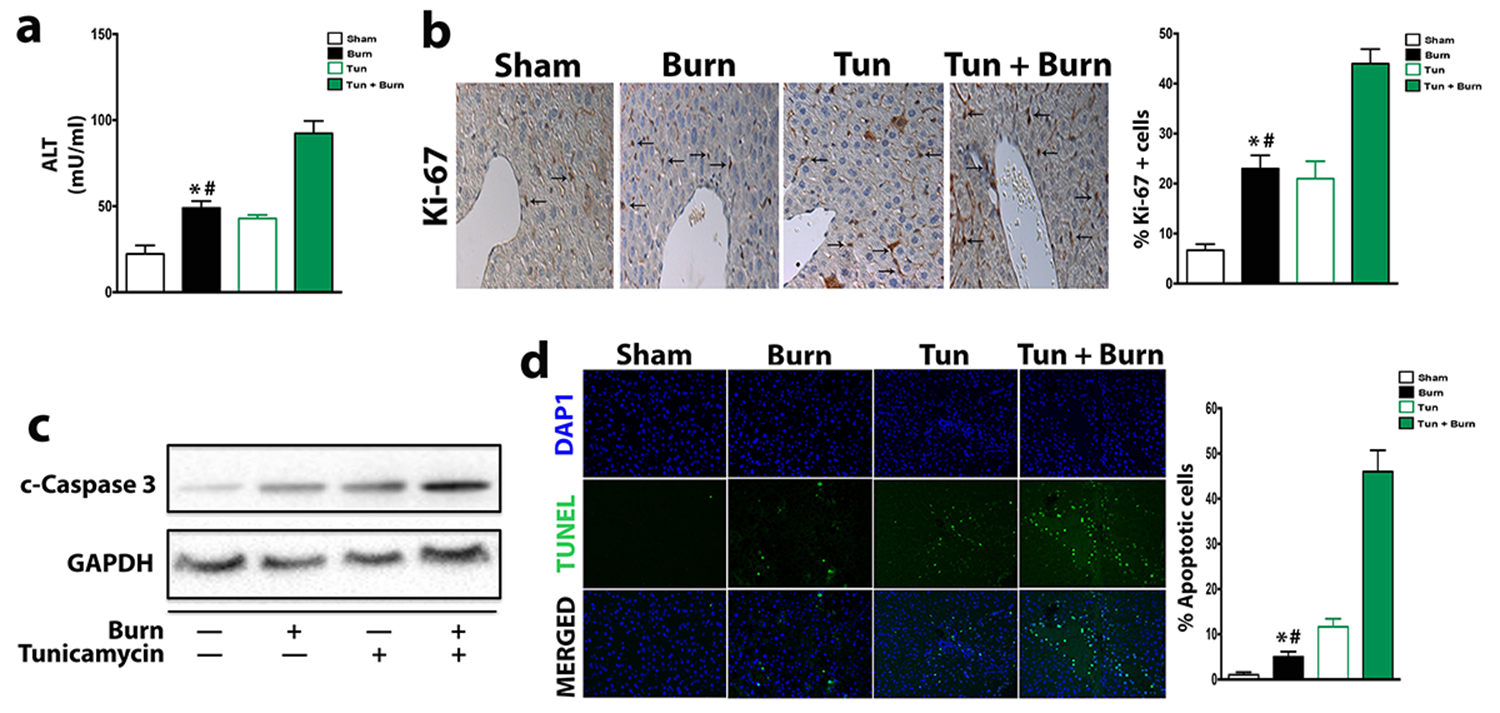

Supplement: Supplementary file 5 — Supplemental Figure 4 [file 41419_2019_2103_MOESM5_ESM.tif]
